# Supplementary material for: Derepression may masquerade as activation in ligand-gated ion channels
Source: Nat Commun. 2023 Apr 5;14:1907. doi: 10.1038/s41467-023-36770-z (PMC10076327; doi:10.1038/s41467-023-36770-z)
Supplement: Supplementary file 1 — Supplementary Information [file 41467_2023_36770_MOESM1_ESM.pdf]

Supplementary Information

Derepression may masquerade as activation in ligand-gated ion channels

Christian J.G. Tessier [1], Johnathon R. Emlaw [1], Raymond M. Sturgeon [1], and Corrie J.B. daCosta [1]✉

[1]Department of Chemistry and Biomolecular Sciences, and Centre for Chemical and Synthetic Biology, University of Ottawa, Ontario, Canada

Correspondence: *cdacosta@uottawa.ca*

Author Contributions:

C.J.G.T. acquired and analyzed all electrophysiological data, while R.M.S. and J.R.E. acquired preliminary α/β_{Anc} heteromer recordings. J.R.E., C.J.B.d.C., and C.J.G.T. performed radiolabelled α-Btx experiments. C.J.G.T. and C.J.B.d.C. interpreted the data and wrote the manuscript. C.J.B.d.C. supervised the project.

Includes:

- Supplementary Fig. 1 to 5

  (6 pages total)

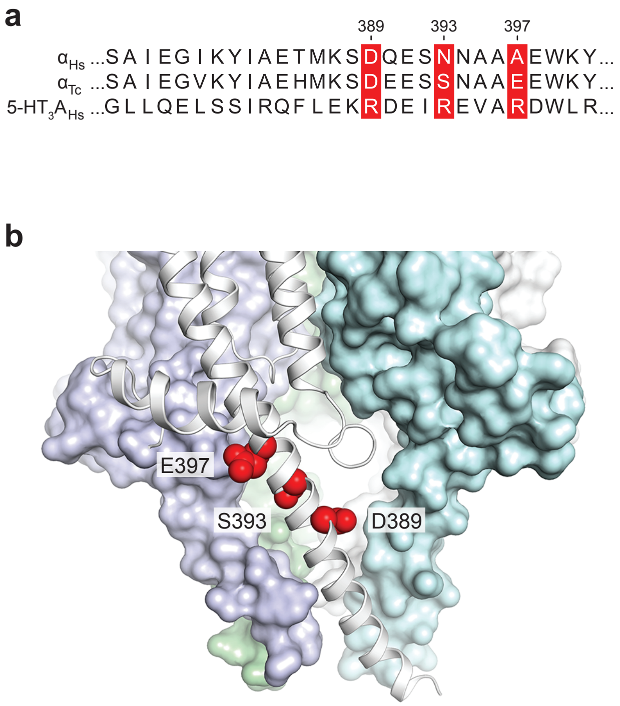

Supplementary Fig. 1. Structure of the acetylcholine receptor cytoplasmic portals. (**a**) Multiple sequence alignment of a select region of the cytoplasmic domains of the human (*Homo sapiens*; Hs) and *Tetronarce californica* (Tc) α-subunits, as well as the human 5-HT3$_A$ subunit. Highlighted in red are the positions corresponding to the three arginine residues known to limit conductance of the 5-HT3$_A$ receptor (ref. 34), which were mutated to arginine in the human α-subunit in the present work to produce the low conductance variant (α_{LC}) . (**b**) Structure of the cytoplasmic region of the *Tetronarce californica* acetylcholine receptor (Protein Data Bank ID: 7QKO; ref. 35). A single α-subunit is shown in cartoon representation (white), while all other subunits are show as molecular surfaces. The three residues that line a cytoplasmic portal, and which in the human α-subunit were mutated to arginine (as in the 5-HT3$_A$ subunit) are shown as red spheres and labelled (D389, S393, and E397).

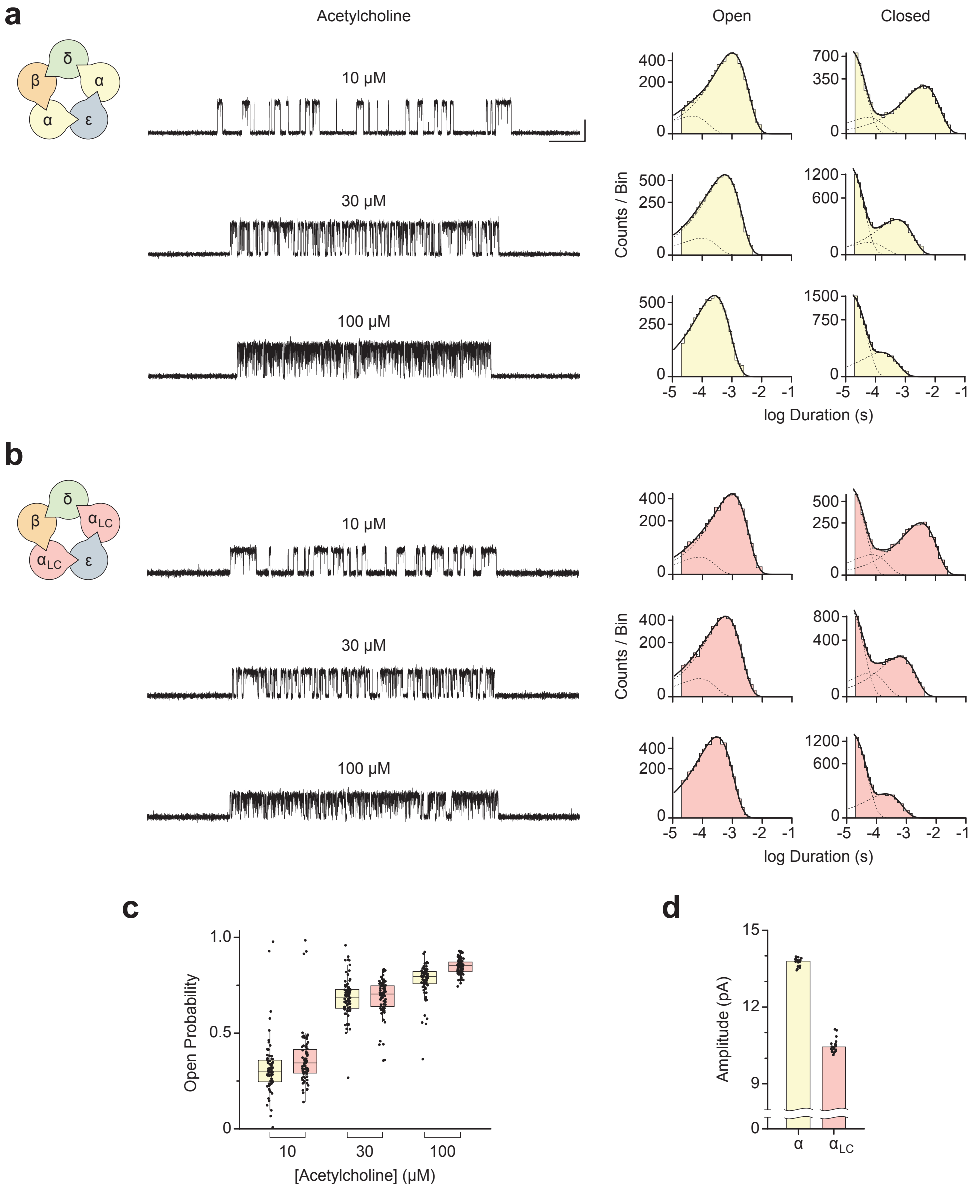

Supplementary Fig. 2. Human adult muscle-type acetylcholine receptors incorporating an α-subunit harbouring conductance mutations (α_{LC}) have a lower single-channel amplitude, but maintain a similar kinetic profile. Subunit composition and single-channel burst activity of (**a**) wild-type and (**b**) α_{LC}-containing human adult muscle-type acetylcholine receptors at the indicated acetylcholine concentrations. In each case, corresponding open and closed duration histograms are shown on the right, and fit (solid line) by a sum of individual exponential components (dashed lines). Recordings were acquired at –120 mV in the cell-attached patch configuration, and digitally filtered with a 10 kHz Gaussian filter. Single-channel openings represent inward cation currents, and are shown as upward deflections. The scale bar in *a* represents 20 ms and 10 pA, and applies to all traces in *a* and *b*. (**c**) Mean open probability for wild-type (yellow) and α_{LC}-containing (rose) channels is similar at the three acetylcholine concentrations. Shown are box-and-whisker plots, where the box spans the 1st and 3rd quartiles and the median is also shown (horizontal line). Whiskers represent 1.5 times the interquartile range. The open probability was determined from a total of 75 bursts (data points) from three different patches (25 bursts each), from two separate transfections. (**d**) Channels harbouring α_{LC} have a reduced single-channel amplitude. Mean channel amplitudes in the presence of 30 μM acetylcholine were determined from a total of 20 bursts from two different patches (10 bursts each), from two separate transfections.

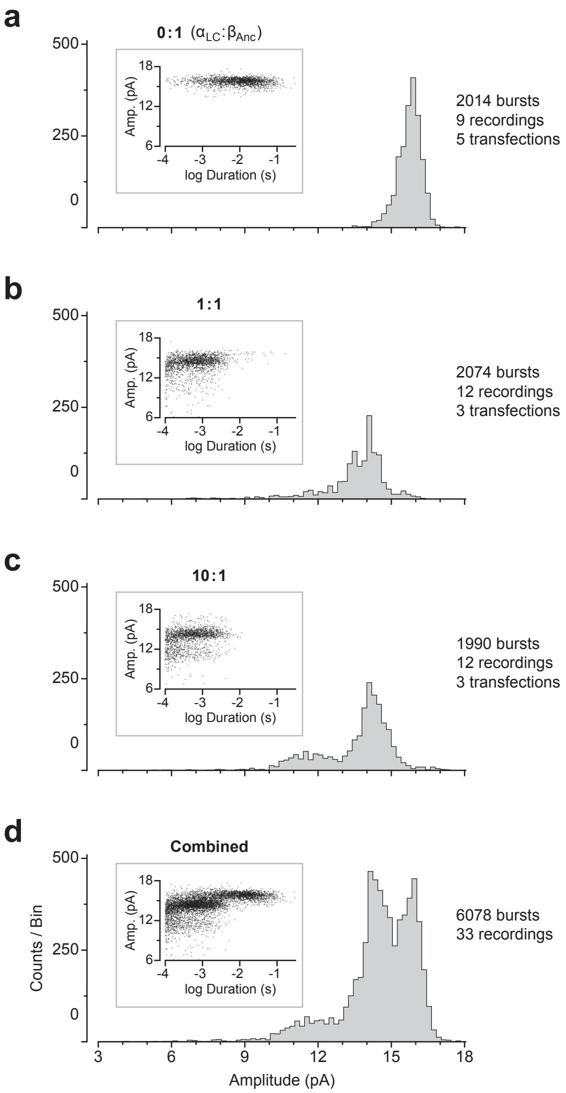

Supplementary Fig. 3. Amplitude distributions for single-channel bursts in patches where cells were transfected with the indicated ratios of α_{LC}:β_{Anc} cDNA. Cells were transfected at (**a**) 0:1, (**b**) 1:1, and (**c**) 10:1 (α_{LC}:β_{Anc} cDNA; by weight). As in Figure 3 of the main text, insets are plots of the amplitude of individual bursts as a function of their duration and reveal an apparent correlation. (**d**) Combining bursts from all three cDNA ratios reveal amplitude classes. As indicated in each panel, each distribution contains approximately 2000 bursts, from 9-12 individual recordings, from between 3-5 separate transfections.

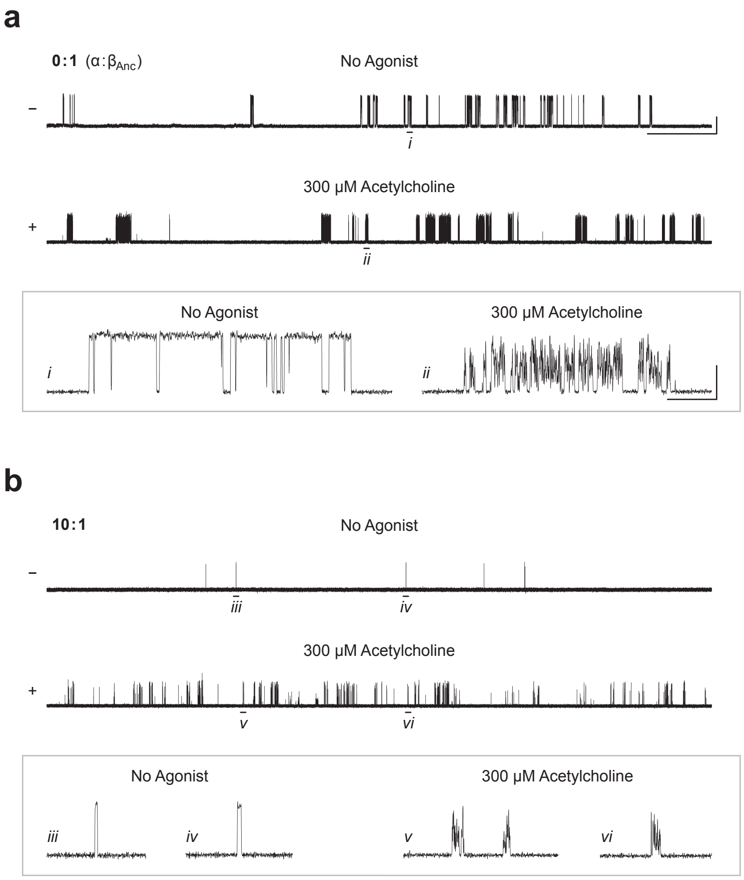

Supplementary Fig. 4. A high concentration of acetylcholine leads to extensive open-channel block. Single-channel activity from cells expressing (**a**) β_{Anc} homopentamers (0:1; by weight; α:β_{Anc}) and (**b**) α/β_{Anc} heteromers (10:1) in the absence (–) and presence (+) of 300 μM acetylcholine. The top two traces in each panel are enlarged version of the traces presented in Figure 4 of the main text, but with select bursts highlighted (*i-vi*, below traces) and enlarged in the boxes below to show the effect of 300 μM acetylcholine. Recordings were obtained in the cell-attached patch configuration with an applied potential of –120 mV and Gaussian filter of 5 kHz. Single-channel openings represent inward cation currents, and are shown as upward deflections. The scale bar beside the top trace in *a* applies to all zoomed out traces and represents 1 s and 10 pA, while the scale bar in the boxed region in *a* represents 10 ms and 10 pA and also applies to the boxed region in *b*.

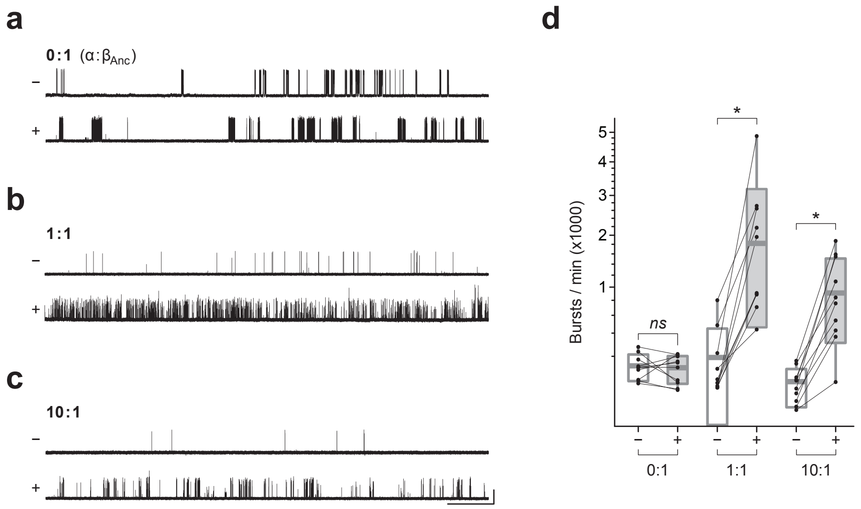

Supplementary Fig. 5. Agonist relieves apparent repression of α/β_{Anc} heteromers (expanded). Figure 4 of the main text has been expanded to include cells transfected at an α:β_{Anc} cDNA ratio of (**b**) 1:1 (by weight). (**a**–**c**) The same cells were patched in the absence (–) and then in the presence (+) of 300 µM acetylcholine. Recordings were obtained in the cell-attached patch configuration with an applied potential of –120 mV and Gaussian filter of 5 kHz. Single-channel openings represent inward cation currents, and are shown as upward deflections. The scale bar beside the bottom trace in *c* applies to all traces and represents 1 s and 10 pA. (**d**) Comparison of burst frequency in paired recordings from cells expressing either β_{Anc} homopentamers (0:1), or α/β_{Anc} heteromers at two different α:β_{Anc} cDNA ratios (1:1 and 10:1). In each case, paired cell-attached recordings from the same cell were acquired first in the absence (–), and then in the presence (+), of 300 µM acetylcholine in the patch pipette. Box plots represent one standard deviation from the mean, with the internal horizontal line denoting the mean of the 10 recordings in each case. Maximum and minimum values are presented as box plot whiskers. As determined by a two-way ANOVA, the difference between mean burst frequency – /+ 300 µM acetylcholine is statistically significant (α level of 0.05) for the α/β_{Anc} heteromers (1:1, $p = 0.0030$; 10:1, $p = 0.0011$; asterisks), but not the β_{Anc} homopentamers (0:1, $p = 0.7189$; *ns*).
