# Supplementary material for: Derepression may masquerade as activation in ligand-gated ion channels
Source: Nat Commun. 2023 Apr 5;14:1907. doi: 10.1038/s41467-023-36770-z (PMC10076327; doi:10.1038/s41467-023-36770-z)
Supplement: Supplementary file 3 — Reporting Summary [file 41467_2023_36770_MOESM3_ESM.pdf]

nature portfolio

Corresponding author(s): Corrie daCosta

Last updated by author(s): Jan 27, 2023

Reporting Summary

Nature Portfolio wishes to improve the reproducibility of the work that we publish. This form provides structure for consistency and transparency in reporting. For further information on Nature Portfolio policies, see our Editorial Policies and the Editorial Policy Checklist.

Statistics

For all statistical analyses, confirm that the following items are present in the figure legend, table legend, main text, or Methods section.

n/a	Confirmed	
☐	☒	The exact sample size (*n*) for each experimental group/condition, given as a discrete number and unit of measurement
☐	☒	A statement on whether measurements were taken from distinct samples or whether the same sample was measured repeatedly
☐	☒	The statistical test(s) used AND whether they are one- or two-sided<br>*Only common tests should be described solely by name; describe more complex techniques in the Methods section.*
☒	☐	A description of all covariates tested
☐	☒	A description of any assumptions or corrections, such as tests of normality and adjustment for multiple comparisons
☐	☒	A full description of the statistical parameters including central tendency (e.g. means) or other basic estimates (e.g. regression coefficient) AND variation (e.g. standard deviation) or associated estimates of uncertainty (e.g. confidence intervals)
☐	☒	For null hypothesis testing, the test statistic (e.g. *F*, *t*, *r*) with confidence intervals, effect sizes, degrees of freedom and *P* value noted<br>*Give P values as exact values whenever suitable.*
☒	☐	For Bayesian analysis, information on the choice of priors and Markov chain Monte Carlo settings
☒	☐	For hierarchical and complex designs, identification of the appropriate level for tests and full reporting of outcomes
☒	☐	Estimates of effect sizes (e.g. Cohen's *d*, Pearson's *r*), indicating how they were calculated

Our web collection on statistics for biologists contains articles on many of the points above.

Software and code

Policy information about availability of computer code

Data collection	Single Channel recordings recorded in Bruxton Acquire (v 6.0.0)
Data analysis	TAC (v 4.3.3; Bruxton) and TACFit (v 4.3.3; Bruxton) were used for single channel analysis and data presentation.<br>GraphPad Prism (v 8.0.0) and R (v 4.0.4) was used for data analysis and statistical tests. R packages used include; ggpubr (v 0.4.0), ggplot2 (v 3.3.5), scbursts (v 1.6), extremevalues (v 2.3.3), MASS (v 7.3-55).<br><br>R scripts for defining and sorting bursts are freely available on figshare (https://doi.org/10.6084/m9.figshare.21742247).

For manuscripts utilizing custom algorithms or software that are central to the research but not yet described in published literature, software must be made available to editors and reviewers. We strongly encourage code deposition in a community repository (e.g. GitHub). See the Nature Portfolio guidelines for submitting code & software for further information.

Data

Policy information about availability of data

All manuscripts must include a data availability statement. This statement should provide the following information, where applicable:

- Accession codes, unique identifiers, or web links for publicly available datasets
- A description of any restrictions on data availability
- For clinical datasets or third party data, please ensure that the statement adheres to our policy

All source data associated with the current study are available in a figshare repository (https://doi.org/10.6084/m9.figshare.21742247). This includes detected single channel openings and closings ('evt' files also in 'txt' format), as well as raw data and any corresponding analysis scripts. Raw single-channel recordings in Bruxton Acquire format (*.acquire format; approximately 1 GB each) are available upon request. Supplementary Fig. 1 was made using Protein Data Bank ID: 7QKO [http://doi.org/10.2210/pdb7QKO/pdb].

Human research participants

Policy information about studies involving human research participants and Sex and Gender in Research.

Reporting on sex and gender	N/A
Population characteristics	N/A
Recruitment	N/A
Ethics oversight	N/A

Note that full information on the approval of the study protocol must also be provided in the manuscript.

Field-specific reporting

Please select the one below that is the best fit for your research. If you are not sure, read the appropriate sections before making your selection.

☒ Life sciences ☐ Behavioural & social sciences ☐ Ecological, evolutionary & environmental sciences

For a reference copy of the document with all sections, see nature.com/documents/nr-reporting-summary-flat.pdf

Life sciences study design

All studies must disclose on these points even when the disclosure is negative.

Sample size	Electrophysiology data for each experiment were obtained from a minimum of 2 different transfections performed on different days. The number of recordings, each from a different cells, is indicated in each case. Sample sizes were set at ~2000 bursts per condition, thereby providing sufficient data points to populate burst duration histograms.
Data exclusions	Single channel electrophysiology recordings were obtained within a noise and single-channel activity threshold. Bursts or sweep where simultaneous opening of multiple channels were omitted, or in some cases the entire recording was not analyzed.
Replication	For single-channel recordings, each replicate represents data acquired from the same cell-attached patch, where each patch was from a different/independent cell. In each case, replicates (minimum of 8 separate recordings) were acquired until a minimum of ~2000 bursts were amassed. For the paired replicates in Figures 4 and S5, consecutive cell-attached patches, first in the absence and then in the presence of 300 µM acetylcholine, were acquired from the same cell. For radioligand binding experiments (with either 2 or 3 independent replicates), each replicate constitutes data acquired from a separate transfection on a different day.
Randomization	All experiments required mammalian cell transfections. All transfections were tracked without randomization.
Blinding	The same analysis procedure was maintained irrespective of transfection conditions. No blinding was used in this study.

Reporting for specific materials, systems and methods

We require information from authors about some types of materials, experimental systems and methods used in many studies. Here, indicate whether each material, system or method listed is relevant to your study. If you are not sure if a list item applies to your research, read the appropriate section before selecting a response.

Materials & experimental systems

n/a	Involved in the study
☒	☐ Antibodies
☐	☒ Eukaryotic cell lines
☒	☐ Palaeontology and archaeology
☒	☐ Animals and other organisms
☒	☐ Clinical data
☒	☐ Dual use research of concern

Methods

n/a	Involved in the study
☒	☐ ChIP-seq
☒	☐ Flow cytometry
☒	☐ MRI-based neuroimaging

Eukaryotic cell lines

Policy information about cell lines and Sex and Gender in Research

Cell line source(s): Cellosaurus BOSC-23 (CVCL_4401), originally from ATCC (ATCC number: CRL-11270; discontinued)

Authentication: STR profiling using Promega's GenePrint® 24 System was performed by The Centre for Applied Genomics Genetic Analysis Facility (The Hospital for Sick Children, Toronto, Canada). A similarity search on the 8,159 human cell lines with STR profiles in Cellosaurus release 42.0 was conducted on the resulting STR profile, which revealed that the cell line shares closest identity (88%, CLASTR 1.4.4 STR Similarity Search Tool score) with Anjou 65 (CVCL_3645). Anjou 65 is a child of CVCL_1926 (HEK293T/17) and is itself a parent line of CVCL_X852 (Bartlett 96). Bartlett 96 is the parent line of BOSC-23 (Pear et al., 1993; Ref. 37).

Mycoplasma contamination: PCR tests confirmed that the cells were free from detectable mycoplasma contamination.

Commonly misidentified lines (See ICLAC register): CVCL_4401 not present in ICLAC.
